# Tracking of anthropometric characteristics from childhood to adolescence: an 8-year follow-up findings from the Czech ELSPAC study

**DOI:** 10.1186/s12889-022-13178-w

**Published:** 2022-04-12

**Authors:** Martin Zvonar, Lovro Štefan, Mario Kasović, Pavel Piler

**Affiliations:** 1 RECETOX, Faculty of Science, Masaryk University, Kotlarska 2, 611 37 Brno, Czech Republic; 2 Department of General and Applied Kinesiology, Faculty of Kinesiology, University of Zagreb, Horvaćanski zavoj 15, 10 000 Zagreb, Croatia; 3 Department of Sport Motorics and Methodology in Kinanthropology, Faculty of Sports Studies, Masaryk University, Kamenice 753/5, 625 00 Brno, Czech Republic

**Keywords:** Obesity, Circumferences, Skinfolds, Indices, Stability, Youth

## Abstract

**Background:**

Although evidence suggests that obesity track well from childhood to adolescence, most of the research has been done in Western and high-income countries. Moreover, most of the studies have tracked body-mass index, as a proxy of nutritional status, while tracking characteristics of circumferences and skinfold thicknesses have been less studies. Therefore, the main purpose of the study was to explore tracking characteristics of complete anthropometric data from childhood to adolescence.

**Methods:**

This sub-study was part of the Czech ELSPAC study. In the present 8-year longitudinal study, we collected information from pediatrician’s medical records at the ages of 8 y (*n* = 888), 11 y (*n* = 1065), 13 y (*n* = 811) and 15 y (*n* = 974), including circumferences (head, chest, waist, hips, and arm), indices (body-mass index, waist-to-hip ratio and waist-to-height ratio) and skinfold thicknesses (biceps, triceps, subscapula, suprailiaca, thigh and the sum of 5 skinfolds). Participants were recruited from the two selected regions of the Czech Republic (Brno and Znojmo). Linear generalized estimating equations were conducted to analyze tracking patterns over an 8-year follow-up period for all anthropometric measurements.

**Results:**

Tracking coefficients were moderate to strong, ranging from 0.40 to 0.62 for circumferences, 0.41 to 0.74 for indices and 0.72 to 0.86 for skinfolds. According to body-mass index and waist circumference standards, overweight/obese children and children with abdominal obesity at the age of 8 y were 11.31 (95% CI = 8.41 to 15.22, *p* < 0.001) and 10.73 (95% CI = 7.93 to 14.52, *p* < 0.001) more likely to remain overweight/obese and to have abdominal obesity at the age of 15 y.

**Conclusions:**

Findings show moderate to strong tracking of anthropometric characteristics, i.e. circumferences track moderately well, while strong tracking for indices and skinfold thicknesses is observed. Moreover, strong tracking of general overweight/obesity and abdominal obesity between ages 8 y and 15 y indicates that the detection of these risk factors at the beginning of primary school should be advocated.

## Background

Childhood obesity has become a global public health challenge and one of the fastest growing non-transmissible problem [1–3]. Estimates suggest that the prevalence of overweight and obesity is between 20 and 45% in European children [4]. Although a recent global analysis has highlighted, that childhood overweight and obesity continue to plateau in high-income countries [3], the rising proportion of overweight and obese children continued to increase in most European countries, with the highest prevalence in middle and southern European countries [5]. In Czech Republic, it has been shown that the prevalence of overweight and obesity appears to rapidly increase, being between 20 and 30% in 11- to 15-year-old children and adolescents [6, 7]. Previous evidence shows, that being overweight or obese in childhood leads to health-related consequences later in life, including premature mortality [8] and higher prevalence of cardiovascular and metabolic diseases [9].

The tracking is most often defined as: (1) “a tendency of individuals to maintain their rank within a certain group over a period of time” [10] and (2) “the ability to predict future observations based on earlier values” [11]. It has been documented, that overweight and obesity (predominantly measured by body-mass index) track well from childhood to adolescence [12–20] and in some studies also into adulthood [21–26]. In general, a systematic review by Singh et al. [24] has shown that overweight and obesity moderately track from childhood to adulthood. However, there is still a paucity of studies investigating tracking of weight status during childhood and adolescence [27]. Also, the comparison of studies is complicated by differences in study design, definition of obesity and analytical methods used [28]. Most of the previous studies have used intraclass [15, 17] or Pearson product-moment correlations [12, 13, 22] and odds/risk ratios [14, 16, 18] to determine the magnitude of tracking during a follow-up period. In recent years, more appropriate statistical methods have been developed and integrated to explore the tracking characteristics of anthropometric data. For instance, only a few studies on the same topic have used generalized estimating equations to calculate a tracking coefficient [16, 29] with a premise that the baseline measurement value is regressed on the entire follow-up development of that specific variable from the other measurements. The obtained beta coefficient is called ‘the tracking coefficient’ and ranges between 0 and 1, with 1 indicating perfect tracking and 0 indicating no tracking. Such method was introduced by Twisk [30] in order to analyze longitudinal data. Generalized estimating equations may be superior statistical methods for calculating the tracking characteristics, since the procedure assumes that the cause of missing data is missing at random and treats missing values as missing [31]. Although much of the research has been conducted in high-income and predominantly Western countries [12], only a limited data are available for middle- or low-income countries and countries from the former Soviet Bloc (like the Czech Republic). Furthermore, to the best of our knowledge, no study has simultaneously investigated the tracking of circumferences, indices and skinfold thicknesses from childhood to adolescence.

The present study focused on the tracking of circumferences (head, chest, waist, hips, and arm), indices (body-mass index, waist-to-hip ratio and waist-to-height ratio) and skinfold thicknesses (biceps, triceps, subscapula, suprailiaca, thigh and the sum of 5 skinfolds) from childhood to adolescence among Czech schoolchildren who entered primary school in the school years 1999 and turned 15 years of age in the school years 2006. Several previous studies have highlighted two critical periods for developing obesity; around the age of 7 y and adolescence [32–34]. Thus, the analysis includes the measurements at the ages of 8 y, 11 y, 13 y and 15 y. We used longitudinal data to investigate: (i) tracking coefficients of circumferences, indices and skinfold thicknesses and (ii) to which extent overweight and obese children at the age of 8 y stay overweight and obese at the age of 15 y.

## Methods

### Study participants

Study data were derived from the Czech part of the ELSPAC study, a prospective birth cohort study carry out in the Czech Republic. The ELSPAC study investigated the effects of biological, psychosocial, economic and environmental factors on pregnancy, delivery and subsequent child’s development and health. The Czech part was approved for adherence to ethical guidelines by the Scientific Council of Masaryk University. The Czech cohort targeted the entire population of births in Brno and Znojmo regions of Czechoslovakia (now Czech Republic) between March 1, 1991, and June 30, 1992. The participation rate of invited pregnant women was 96%. All participating women provided written informed consent. The mother–child pairs were followed mainly by health records, examinations, and self—reported questionnaires completed by parents and later on by the children themselves. A total of 4811 mothers completed first self-reported postnatal questionnaires. Additional details about the study have been published earlier [35]. We based our data on pediatrician’s medical assessments at the ages of 8 y (*n* = 888), 11 y (*n* = 1065), 13 y (*n* = 811) and 15 y (*n* = 974). Pediatrician’s medical records included height, weight, circumferences (head, chest, waist, hips and arm) and skinfolds thicknesses (biceps, triceps, subscapula, suprailiaca and thigh). Prior the study, the pediatricians who evaluated the children were all instructed of how to conduct the anthropometrical measurements. During the project, the same pediatricians were able to measure the children, according to their place of residence. In addition, socioeconomic status of the participants was represented by a one-item variable including maternal educational level at the time of pregnancy with three categories: 1) elementary, 2) secondary and 3) university.

### Anthropometric data

Height and weight were objectively measured using stadiometer and digital scale with a precision of 0.1 cm and 0.1 kg. Circumferences were measured using a non-stretch (plastic) measurement tape (in cm) as follows:

1. **Head**—the tape was placed on the widest part of the head, 1 cm above the eyebrows.
2. **Chest**—the tape was placed on the skin at the level of the middle of the sternum (breastbone), passing under the relaxed arms in the position parallel to the floor. The result was recorded at the end of a normal expiration.
3. **Waist**—the tape was placed horizontally midway between the lower rib margin and the iliac crest at the end of normal expiration for each participant while standing still.
4. **Hips** – while the participant was standing still with the arm relaxed at the side, the tape was placed at the widest level parallel to the floor at the point of measurement, over the trochanters.
5. **Arm** – the tape was placed parallel to the floor at the mid-point between the bony protrusion on the shoulder and the point of the elbow.

Indices included body-mass index, waist-to-hip ratio and waist-to-height ratio. Body-mass index was calculated by dividing weight in kg with height in m^2^ [weight (kg)/height(m)^2^]. To define overweight/obese according to body-mass index and waist circumference, the 85^th^ and the 95^th^ body-mass index and waist circumference for age, the Centers for Disease Control and Prevention reference percentiles were used.^36^ Waist-to-hip and waist-to-height ratios were calculated as waist circumference (in cm) divided by the hip circumference (in cm) and height (in cm).

Skinfold thicknesses were measured using John Bull skinfold caliper (in mm) as follows:

1. **Biceps**—the landmark was located at the level of the mid-point between the lateral edge of the acromion process and the radiale (elbow joint), on the mid-line of the front surface of the arm. The arm was relaxed with the palm of the hand facing forward.
2. **Triceps**—the landmark was located at the level of the mid-point between the lateral edge of the acromion process and the radiale (elbow joint), on the mid-line of the back surface of the arm. The arm was relaxed with the palm of the hand facing forward.
3. **Subscapula**—the landmark was placed at the lower angle of the scapula, and the natural fold of the skin was pinched at about 45 degrees.
4. **Suprailiaca** – the landmark was placed at the intersection between the spinale and the horizontal line at the level of the iliac crest.
5. **Thigh** – the landmark was placed at the midpoint of the surface of the thigh, between the patella and the crease at the top of the thigh.
6. **Sum of 5 skinfolds** – the sum of biceps, triceps, subscapula, suprailiaca and thigh skinfold thicknesses.

### Data analysis

Basic descriptive statistics of the study participants are presented as mean and standard deviation (SD). To reduce the skewness of the skinfold thickness distributions, we log-transformed all skinfold variables. Differences between untransformed and log-transformed were only trivial (-0.12 to 0.14), so the data for untransformed variables are presented. Tracking of circumferences, indices and skinfold thicknesses at the ages of 8 y, 11 y, 13 y and 15 y was assessed using linear generalized estimating equations with exchangeable working correlation matrix structure in all analyses. To describe the magnitude of tracking, the baseline values (the age of 8 y) were regressed on the entire follow-up data from the age of 11 y to the age of 15 y. The obtained beta coefficient is interpreted as the tracking coefficient [30]. The coefficient ranges between 0 and 1, where 1 indicates perfect tracking and 0 indicates no tracking. To evaluate the tracking of overweight/obesity over an 8-year follow-up period, we used odds ratios (ORs) with 95% confidence intervals (95% CI) derived from the binary logistic generalized estimating equations. For the purpose of tracking overweight/obesity and abdominal obesity (derived from body-mass index and waist circumference), participants were grouped into normal weight (< 85^th^ percentile) and overweight/obesity (≥ 85^th^ percentile) [36]. Body-mass index and waist circumference categories (< 85^th^–normal weight, 85^th^- < 95^th^ overweight and ≥ 95^th^–obesity) were cross-tabulated and the percentages are shown for each year of measurement. In general, no significant differences were found in the study variables between sexes, so the sex-specific analyses were not included in our final models. Two-sided *p*-values were used, and significance was set at α < 0.05. All the analyses were calculated in Statistical Packages for Social Sciences v.23 (SPSS, Chicago, IL, United States).

## Results

The number of children involved in all 8-year measurements changed slightly, including 888 at the age of 8 y; 1065 at the age of 11 y; 811 at the age of 13 y; and ended with 974 at the final assessment. Average values for circumferences, indices and skinfold thicknesses are presented in Table 1. Values for circumferences gradually increased, while an increase, a stagnation or a decrease for other study variables during the follow-up period were observed. Of note, most mothers completed secondary education (35.0%), followed by primary education (28.4%) and university (16.2%). Approximately 20.0% of collected questionnaires had missing values.

**Table 1:** Descriptive statistics of the study participants

Study variables	Year 8	Year 11	Year 13	Year 15	*p*-value
**Circumferences**	Mean (SD)	Mean (SD)	Mean (SD)	Mean (SD)	
Head (cm)	52.1 (2.3)	53.8 (1.7)	54.4 (2.5)	54.6 (3.8)	< 0.001
Chest (cm)	62.2 (4.6)	72.1 (6.7)	79.3 (6.8)	84.1 (6.0)	< 0.001
Waist (cm)	57.9 (5.8)	63.8 (7.2)	69.8 (7.7)	70.5 (6.9)	< 0.001
Hips (cm)	66.6 (5.1)	77.9 (7.3)	86.5 (8.2)	91.3 (6.6)	< 0.001
Arm (cm)	19.8 (2.0)	21.9 (2.6)	23.8 (4.3)	25.0 (4.6)	< 0.001
**Indices**					
Body-mass index (kg/m^2^)	16.1 (2.0)	17.4 (2.7)	19.1 (2.9)	20.1 (2.6)	< 0.001
Waist-to-hip ratio	0.87 (0.05)	0.82 (0.05)	0.81 (0.08)	0.78 (0.1)	< 0.001
Waist-to-height ratio	0.44 (0.04)	0.42 (0.04)	0.43 (0.04)	0.41 (0.04)	< 0.001
**Skinfold thicknesses**					
Biceps (mm)	7.8 (3.3)	7.3 (3.5)	6.8 (3.8)	5.9 (2.8)	< 0.001
Triceps (mm)	10.7 (4.6)	11.2 (4.7)	12.4 (5.3)	12.3 (5.1)	< 0.001
Subscapula (mm)	8.9 (4.9)	9.1 (5.3)	9.2 (5.1)	9.3 (4.2)	0.507
Suprailiaca (mm)	9.9 (5.8)	9.3 (6.0)	8.7 (5.6)	8.1 (4.5)	< 0.001
Thigh (mm)	20.5 (8.6)	20.2 (8.1)	19.6 (9.1)	19.8 (9.7)	0.416
Sum5skinfolds (mm)	57.8 (24.4)	57.1 (23.4)	56.7 (25.0)	55.4 (24.2)	0.163

Table 2 shows tracking coefficients for circumferences, indices and skinfold thicknesses derived from generalized estimating equations. In general, tracking coefficients were moderate to high for circumferences, mostly high for indices and high for skinfold thicknesses. Specifically, tracking coefficients for circumferences ranged between 0.40–0.62, with the lowest tracking being observed for chest and hips and the strongest tracking being observed for head. The corresponding tracking coefficients for indices ranged between 0.41–0.74, and for skinfold thicknesses between 0.72–0.86.

**Table 2:** Tracking coefficients for anthropometric circumferences, indices and skinfold thicknesses at the ages of 8 y, 11 y, 13 y and 15 y

Study variables	Tracking coefficient	95% CI	Standard error	*p*-value
**Circumferences**				
Head (cm)	0.62	0.50 to 0.74	0.06	< 0.001
Chest (cm)	0.40	0.36 to 0.43	0.02	< 0.001
Waist (cm)	0.59	0.56 to 0.62	0.02	< 0.001
Hips (cm)	0.40	0.37 to 0.43	0.02	< 0.001
Arm (cm)	0.44	0.36 to 0.55	0.05	< 0.001
**Indices**				
Body-mass index (kg/m^2^)	0.67	0.64 to 0.70	0.01	< 0.001
Waist-to-hip ratio	0.41	0.29 to 0.57	0.08	< 0.001
Waist-to-height ratio	0.74	0.68 to 0.79	0.03	< 0.001
**Skinfolds**				
Biceps (mm)	0.78	0.67 to 0.87	0.05	< 0.001
Triceps (mm)	0.81	0.78 to 0.83	0.01	< 0.001
Subscapula (mm)	0.86	0.84 to 0.88	0.01	< 0.001
Suprailiaca (mm)	0.81	0.78 to 0.83	0.01	< 0.001
Thigh (mm)	0.72	0.68 to 0.75	0.02	< 0.001
Sum5skinfolds (mm)	0.83	0.81 to 0.85	0.01	< 0.001

**Fig. 1:**
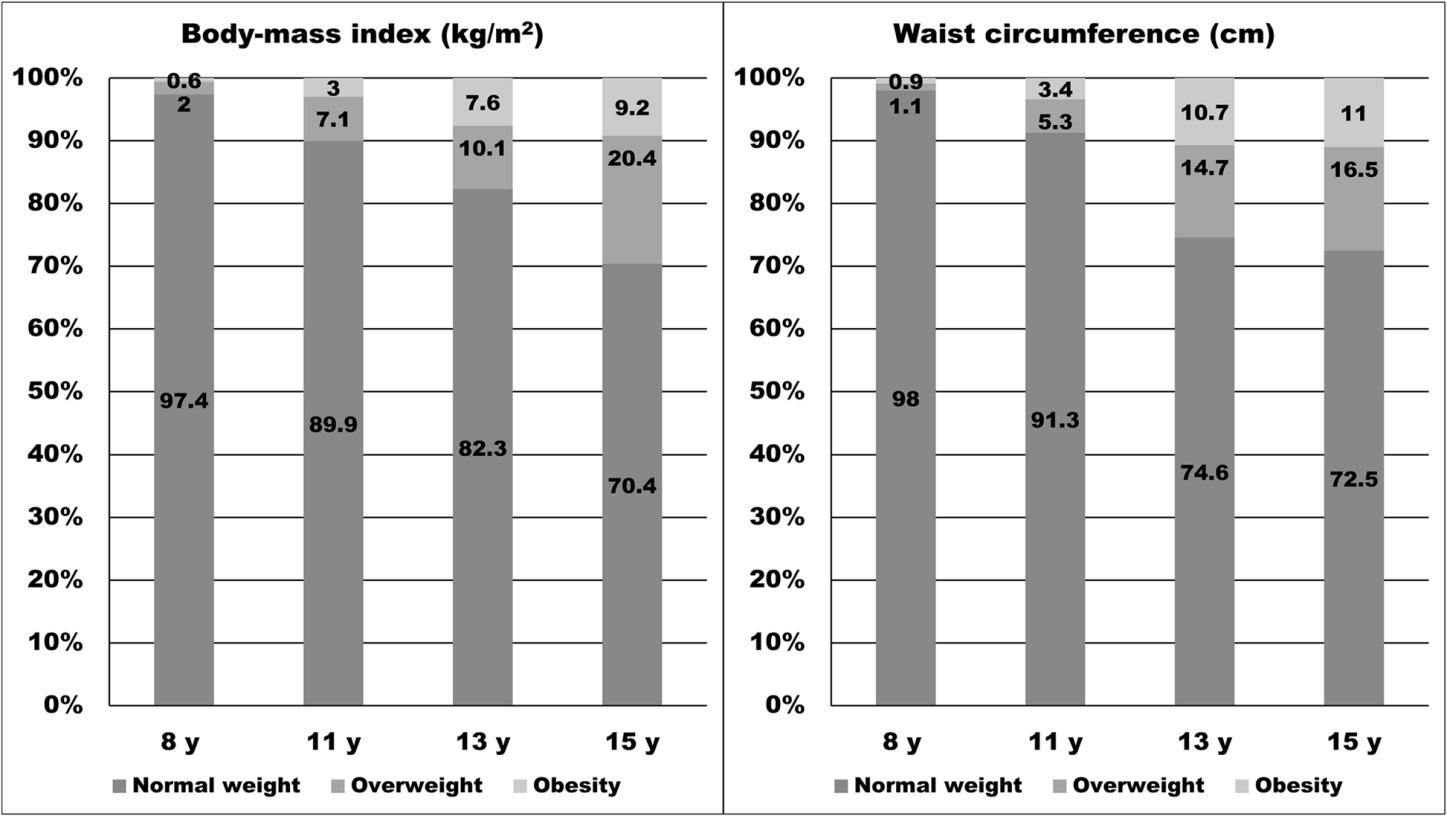
Tracking of overweight/obesity over 8-year follow-up period

Figure 1 illustrates the tracking of overweight and obesity grouped from body-mass index and abdominal obesity derived from waist circumference. The general trend was that the higher body-mass index or waist circumference group at the age of 8 y, the higher was the probability of being overweight/obese and having abdominal obesity at the age of 15 y. The chances of an overweight and obese 8-year-old becoming an overweight or obese 15-year-old increased consistently with age. Children, who were overweight/obese derived from body-mass index at the age of 8 y had 11.31 (95% CI = 8.41 to 15.22, *p* < 0.001) more odds of remaining overweight/obese throughout the age of 15 y, compared to their peers, respectively. Similar odds were obtained for abdominal obesity, where those children with waist circumference ≥ 85^th^ percentile, indicating abdominal obesity, were 10.73 (95% CI = 7.93 to 14.52, *p* < 0.001) more likely to remain having abdominal obesity at the age of 15 y.

## Discussion

The main purpose of the study was to explore tracking characteristics of anthropometric data in an 8-year follow-up study. The main findings are: (i) circumferences and indices show moderate to high tracking, while the tracking of skinfold thicknesses was higher; (ii) overweight and obese 8-year-old children are roughly 11 times more likely to remain overweight and obese at the age of 15 y, compared to their normal weight peers; and (iii) a significant increase in the proportion of overweight and obese individuals from the age of 8 y to the age of 15 y is observed.

To understand the context of main findings, social and cultural background of Czech Republic is to be highlighted. It has been well-established, that tracking childhood overweight and obesity beyond adult age represents a major limitation of the literature [12], but there is still a considerable lack of studies from developing countries experiencing major socio-economical changes [34]. The Czech Republic is the country that has been experiencing transitive changes, including lifestyle and nutritional habits [7]. Children and youth from low- and middle-income European countries tend to repeat behavioral patterns from the Western high-income countries, which consequently leads to increased rates of overweight and obesity [7]. Such patterns have been observed in Czech Republic, where excessive screen time and insufficient physical activity significantly increase the change of overweight/obesity in later years [37].

Tracking overweight and obesity from childhood to adolescence has been mainly the topic of Western and high-income countries, including Australia [14, 27], United States of America [22], Norway [16, 19], Finland [13], Iceland [20] and some middle-income countries, like Brazil [15, 17] China [18] and Slovenia [12]. In general, body-mass index track well from childhood to adolescence, and even to adulthood [24]. Our result of tracking body-mass index (*beta regression coefficient* = 0.67) is similar to one previous study on the same topic [12]. The findings from a 12-year follow-up study in Slovenia showed high tracking of body-mass index from the age of 7 y to the age of 14 y (*r* = 0.71 and 0.66 in boys and girls, respectively) [12]. However, previous evidence has presented larger tracking coefficients [15, 17], compared to our results. The two Brazilian studies showed extremely high tracking characteristics of body-mass index of 0.94 [15] and 0.87 [17]. The discrepancy between the studies may be explained by methodological differences, that is, children in studies by Ronque et al. [15] and Prado et al. [17] were followed-up for 3 y and 4 y, while our follow-up period was longer (8 y). Also, the aforementioned studies used intraclass correlation coefficients to calculate the tracking, while we used generalized estimating equations with *beta* coefficient. Finally, sociocultural and economic changes within the countries might be responsible for different results. This statement can be confirmed by the results from the Slovenian study [12]. For instance, Slovenia and Czech Republic are both central European countries with similar history backgrounds and lifestyle patterns, which influence physical development of children and youth [12].

We also found high tracking coefficients for skinfold thicknesses, which is similar to one previous study conducted among Brazilian schoolchildren [15]. In the study by Ronque et al. [15], intraclass correlation coefficients were 0.85 for triceps skinfold thickness, 0.84 for subscapula skinfold thickness and 0.86 for the sum of triceps and subscapular skinfold thicknesses. Similar results were reported previously [38, 39]. Findings from another study showed somewhat smaller tracking coefficients for triceps (0.51–0.76), biceps (0.56–0.73), subscapula (0.55–0.64) and suprailiaca (0.63 to 0.74) skinfold thicknesses [29]. Finally, tracking coefficients for triceps skinfold thickness were only moderate (0.52 and 0.49 for boys and girls, respectively) in one Slovenian study [12]. In the study by Monyeki et al. [29], a relatively high prevalence of thinness in primary school-aged children (23.7–30.0%) might have resulted in lower tracking, especially because of irregular appetite control or energy maintenance functions [40].

Finally, we found that overweight/obese children at the age of 8 y were more likely to be overweight/obese at the age of 15 y, which was confirmed by previous studies [12, 28]. It has been reported that about one-third of overweight and obese children remained overweight and obese later in life [12]. Our results showed, that about 30.0% of adolescents aged 15 y were overweight or obese at the age of 8 y. High proportion of overweight and obesity can partially be explained by a different environment future adolescents lived in, regarding of fat-rich and fast food, physical inactivity and higher prevalence of sedentary behaviors.

This study has several strengths. The findings presented in this paper may be used for comparative studies. Central European population of children born in the early 1990s grew up under specific socioeconomic and cultural conditions, which were very different from those in Western part of Europe [41]. A longitudinal design allows us to establish a certain causality between several time-points, and to observe changes in a given variable. Also, objective measurements for all anthropometric data by a trained pediatrician lower the chance of measurement error and potential bias.

However, this study is not without limitations. First, biological samples of children were not assessed at the baseline. Second, the models in the study were not adjusted for environmental factors, including physical activity, dietary patterns, sedentary behaviors and maturational status.

## Conclusions

This study shows moderate to high tracking coefficients for a set of anthropometric characteristics during an 8-year follow-up period between childhood and adolescence. The findings of the present study suggest that the association of anthropometric data from childhood to adolescence will continue to be a major interest to special intervention- and strategy-makers, in order to detect a high-risk group of children and adolescence. Also, a special emphasize needs to be put in tracking overweight and obese children, because children with normal weight have more odds for retaining normal weight in later years. Therefore, these critical periods between childhood and early adolescence should be a cornerstone for school-based programs, aiming to increase the level of physical activity and to decrease the time spent in sedentary behaviors.

## Data Availability

The datasets generated and/or analysed during the current study are not publicly available due to limitations of ethical approval involving the patient data and anonymity but are available from the corresponding author on reasonable request.

## Abbreviations

OR: Odds ratio
95% CI: 95 Percent confidence interval
Sum5skinfolds: Sum of 5 skinfolds

## References

[1] Twig G, Yaniv G, Levine H (2016). Body-mass index in 2.3 million adolescents and cardiovascular death in adulthood. N Engl J Med..

[2] Park MH, Falconer C, Viner RM, Kinra S (2012). The impact of childhood obesity on morbidity and mortality in adulthood: a systematic review. Obes Rev.

[3] NCD Risk Factor Collaboration (NCD-RisC). Worldwide trends in body-mass index, underweight, overweight, and obesity from (1975). to 2016: a pooled analysis of 2416 population-based measurement studies in 128·9 million children, adolescents, and adults. Lancet.

[4] Garrido-Miguel M, Cavero-Redondo I, Álvarez-Bueno C, Rodríguez-Artalejo F, Moreno LA, Ruiz JR (2019). Prevalence and trends of overweight and obesity in European childrenfFrom 1999 to 2016: a systematic review and meta-analysis. JAMA Pediatr..

[5] World Health Organization (2017). Adolescent obesity and related behaviours: trends and inequalities in the WHO European Region, 2002–2014: observations from the Health Behavior in School-Aged Children (HBSC) WHO Collaborative cross-national study.

[6] Hamřík Z, Sigmundová D, Pavelka J, Kalman M, Sigmund E (2017). Trends in overweight and obesity in Czech schoolchildren from 1998 to 2014. Cent Eur J Public Health.

[7] Sigmund E, Sigmundová D, Badura P, Voráčová J, Vladimír H, Hollein T (2020). Time-trends and correlates of obesity in Czech adolescents in relation to family socioeconomic status over a 16-year study period (2002–2018). BMC Public Health.

[8] Lindberg L, Danielsson P, Persson M, Marcus C, Hagman E (2020). Association of childhood obesity with risk of early all-cause and cause-specific mortality: A Swedish prospective cohort study. PLoS Med..

[9] Reilly JJ, Kelly J (2011). Long-term impact of overweight and obesity in childhood and adolescence on morbidity and premature mortality in adulthood: systematic review. Int J Obes (Lond).

[10] Malina RM (1996). Tracking of physical activity and physical fitness across the lifespan. Res Q Exerc Sport.

[11] Foulkes MA, Davis CE (1981). An index of tracking for longitudinal data. Biometrics.

[12] Starc G, Strel J (2011). Tracking excess weight and obesity from childhood to young adulthood: a 12-year prospective cohort study in Slovenia. Public Health Nutr.

[13] Fuentes RM, Notkola IL, Shemeikka S, Tuomilehto J, Nissinen A (2003). Tracking of body mass index during childhood: a 15-year prospective population-based family study in eastern Finland. Int J Obes Relat Metab Disord.

[14] Hayes AJ, Carrello JP, Kelly PJ, Killedar A, Baur LA (2021). Looking backwards and forwards: tracking and persistence of weight status between early childhood and adolescence. Int J Obes.

[15] Ronque ERV, Werneck AO, Bueno MRO, Cyrino ES, Stanganelli LCR, Arruda M (2018). Tracking of body adiposity indicators from childhood to adolescence: mediation by BMI. PLoS One..

[16] Evensen E, Emaus N, Kokkvoll A, Wilsgaard T, Furberg AS, Skeie G (2017). The relation between birthweight, childhood body mass index, and overweight and obesity in late adolescence: a longitudinal cohort study from Norway, The Tromsø Study. Fit Futures. BMJ Open..

[17] Prado TGD, Costa JCD, Bueno MRDO, Batista MB, Romanzin M, Ronque ERV (2018). Tracking of nutritional status between childhood and adolescence in schoolchildren. Rev Bras Med Esporte.

[18] Wang Y, Ge K, Popkin BM (2000). Tracking of body mass index from childhood to adolescence: a 6-y follow-up study in China. Am J Clin Nutr.

[19] Evensen E, Wilsgaard T, Furberg AS, Skeie G (2016). Tracking of overweight and obesity from early childhood to adolescence in a population-based cohort - the Tromsø Study. Fit Futures BMC Pediatr.

[20] Johannsson E, Arngrimsson SA, Thorsdottir I, Sveinsson T (2006). Tracking of overweight from early childhood to adolescence in cohorts born 1988 and 1994: overweight in a high birth weight population. Int J Obes.

[21] Rundle AG, Factor-Litvak P, Suglia SF, Susser ES, Kezios KL, Lovasi GS (2020). Tracking of obesity in childhood into adulthood: effects on body mass index and fat mass index at age 50. Child Obes.

[22] Deshmukh-Taskar P, Nicklas TA, Morales M, Yang SJ, Zakeri I, Berenson GS (2006). Tracking of overweight status from childhood to young adulthood: the Bogalusa Heart Study. Eur J Clin Nutr.

[23] Simmonds M, Burch J, Llewellyn A, Griffiths C, Yang H, Owen C (2015). The use of measures of obesity in childhood for predicting obesity and the development of obesity-related diseases in adulthood: a systematic review and meta-analysis. Health Technol Assess Winch Engl.

[24] Singh AS, Mulder C, Twisk JWR, van Mechelen W, Chinapaw MJM (2008). Tracking of childhood overweight into adulthood: a systematic review of the literature. Obes Rev.

[25] The NS, Suchindran C, North KE, Popkin BM, Gordon-Larsen P (2010). Association of adolescent obesity with risk of severe obesity in adulthood. JAMA.

[26] Juhola J, Magnussen CG, Viikari JSA, Kähönen M, Hutri-Kähönen N, Jula A (2011). Tracking of serum lipid levels, blood pressure, and body mass index from childhood to adulthood: the Cardiovascular Risk in Young Finns Study. J Pediatr.

[27] Wheaton N, Millar L, Allender S, Nichols M (2015). The stability of weight status through the early to middle childhood years in Australia: a longitudinal study. BMJ Open..

[28] Serdula MK, Ivery D, Coates RJ, Freedman DS, Williamson DF, Byers T (1993). Do obese children become obese adults? A review of the literature. Prev Med.

[29] Monyeki KD, Kemper HC, Makgae PJ (2009). Development and tracking of central patterns of subcutaneous fat of rural South African youth: Ellisras longitudinal study. BMC Pediatr.

[30] Twisk JWR. Applied longitudinal data analysis for epidemiology. A practical guide. New York: Cambridge: Cambridge University Press; 2003.

[31] McPherson S, Barbosa-Leiker C, McDonell M, Howell D, Roll J (2013). Longitudinal missing data strategies for substance use clinical trials using generalized estimating equations: an example with a buprenorphine trial. Hum Psychopharmacol.

[32] Stettler N, Zemel BS, Kumanyika S, Stallings VA (2002). Infant weight gain and childhood overweight status in a multicenter, cohort study. Pediatrics.

[33] Dietz WH (1994). Critical periods in childhood for the development of obesity. Am J Clin Nutr.

[34] Williams S, Davie G, Lam F (1999). Predicting BMI in young adults from childhood data using two approaches to modelling adiposity rebound. Int J Obes Relat Metab Disord.

[35] Piler P, Kandrnal V, Kukla L, Andrýsková L, Švancara J, Jarkovský J (2017). Cohort Profile: The European Longitudinal Study of Pregnancy and Childhood (ELSPAC) in the Czech Republic. Int J Epidemiol.

[36] Kuczmarski RJ, Ogden CL, Guo SS, Grummer-Strawn LM, Flegal KM, Mei Z, Wei R, Curtin LR, Roche AF, Johnson CL (2002). 2000 CDC Growth Charts for the United States: methods and development. Vital and health statistics..

[37] Sigmund E, Sigmundová D, Badura P, Kalman M, Hamrik Z, Pavelka J (2015). Temporal trends in overweight and obesity, physical activity and screen time among Czech adolescents from 2002 to 2014: a national health behaviour in school-aged children study. Int J Environ Res Public Health.

[38] van Lenthe FJ, Kemper HCG, Mechelen W, Twisk JWR (1996). Development and tracking of central pattern of subcutaneous fat in adolescence and childhood: The Amsterdam Growth and Health Study. Int J Epidemiol.

[39] Mahoney LT, Lauer RM, Lee J, Clarke WR (1991). Factors affecting tracking of coronary heart disease risk factors in children. The Muscatine Study. Ann N Y Acad Sci..

[40] Cameron N, Demerath EW (2002). Critical period in human growth and their relationship to diseases of aging. Am J Phys Anthropol.

[41] Bobak M, Marmot M (1996). East-West mortality divide and its potential explanations: Proposed research agenda. BMJ.

